# Botulinum toxin A injection for post-stroke upper limb spasticity and rehabilitation practices from centers across Asian countries

**DOI:** 10.3389/fneur.2024.1335365

**Published:** 2024-04-08

**Authors:** Raymond L. Rosales, Nicholas V. C. Chia, Witsanu Kumthornthip, Khean Jin Goh, Choon Soon Mak, Keng He Kong, Yee Sien Ng, Li Wei Chou, M. Jeanne Flordelis, Thuy Do, Pascal Maisonobe, Leonard S. W. Li, Areerat Suputtitada

**Affiliations:** ^1^Department of Neurology and Psychiatry, University of Santo Tomas, Manila, Philippines; ^2^Department of Neuroscience and Brain Health, Center for Neurodiagnostic and Therapeutic Service, Metropolitan Medical Center, Manila, Philippines; ^3^Ipsen, Singapore, Singapore; ^4^Faculty of Medicine Siriraj Hospital, Mahidol University, Bangkok, Thailand; ^5^Division of Neurology, University of Malaya Medical Centre, Kuala Lumpur, Malaysia; ^6^Hospital Kuala Lumpur, Kuala Lumpur, Malaysia; ^7^Tan Tock Seng Hospital, Singapore, Singapore; ^8^Singapore General Hospital, Singapore, Singapore; ^9^China Medical University Hospital, Taichung, Taiwan; ^10^Perpetual Succor Hospital, Cebu City, Philippines; ^11^Ipsen, Ho Chi Minh City, Vietnam; ^12^Ipsen, Boulogne-Billancourt, France; ^13^Tung Wah Hospital, Hong Kong, Hong Kong SAR, China; ^14^Department of Rehabilitation Medicine, Faculty of Medicine, Chulalongkorn University, Bangkok, Thailand; ^15^King Chulalongkorn Memorial Hospital, The Thai Red Cross Society, Bangkok, Thailand

**Keywords:** Asia, botulinum toxin, post-stroke, spasticity, upper limb

## Abstract

**Purpose:**

Describe real-life practice and outcomes in the management of post-stroke upper limb spasticity with botulinum toxin A (BoNT-A) in Asian settings.

**Methods:**

Subgroup analysis of a prospective, observational study (NCT01020500) of adult patients (≥18 years) with post-stroke upper limb spasticity presenting for routine spasticity management, including treatment with BoNT-A. The primary outcome was goal attainment as assessed using goal-attainment scaling (GAS). Patients baseline clinical characteristics and BoNT-A injection parameters are also described.

**Results:**

Overall, 51 patients from Asia were enrolled. Rates of comorbid cognitive and emotional problems were relatively low. Patients tended to have more severe distal limb spasticity and to prioritize active over passive function goals. Most (94.1%) patients in the subgroup were treated with abobotulinumtoxinA. For these patients, the median total dose was 500 units, and the most frequently injected muscles were the biceps brachii (83.3%), flexor carpi radialis (72.9%), and flexor digitorum profundus (66.7%). Overall, 74.5% achieved their primary goal and the mean GAS T score after one treatment cycle was 56.0 ± 13.0, with a change from baseline of 20.9 ± 14.3 (*p* < 0.001). The majority (96.1%) of Asian patients were rated as having improved.

**Conclusion:**

In the Asian treatment setting, BoNT-A demonstrated a clinically significant effect on goal attainment for the real-life management of upper limb spasticity following stroke.

## 1 Introduction

Stroke is one of the leading causes of disability-adjusted life-years in Asian people aged over 50 (1). One of the major contributors to this global disability is spasticity, which has considerable impact on daily activities and patient and caregiver quality of life (2, 3). The role of botulinum neurotoxin A (BoNT-A) as a first-line treatment for focal spasticity is well embedded in national and international guidelines across the world (4, 5). Such recommendations are based on high quality evidence from randomized controlled trials (5, 6), but these are often conducted in restricted populations and do not consider the diversity of patient presentation, nor the varied clinical approaches to treatment used in different countries. For example, different cultures may have different goals for treatment and each country will have their own national approach to care (7). Moreover, BoNT-A injection practices often depend on the injector training available (8), and consequently there is significant international variation in the spasticity-assessment tools used as well in muscle selection, dosing and other clinical parameters.

Epidemiological and treatment data for post-stroke spasticity in Asian populations is relatively sparse. One study, conducted at a single expert rehabilitation clinic in Singapore, found the frequency of symptomatic spasticity was 30% in stroke survivors who required in-patient rehabilitation (9). Otherwise, there is little to no information about what treatments doctors from Asia use in real-life clinical practice. The ULIS-II study was a large, observational, prospective study designed to describe current practice and evaluate outcomes in the routine treatment of post stroke upper-limb spasticity after one cycle of BoNT-A. Uniquely, the multicenter study was conducted in specialist centers from 22 countries from all over the world including six from Asia (10).

In order to further our understanding of the significant gaps in epidemiological and treatment data for post-stroke spasticity in Asian populations, we present here a *post-hoc,* regional (Asian) analysis of ULIS-II outcomes, which included 51 patients from centers spanning Hong Kong, Philippines, Thailand, Taiwan, Malaysia, and Singapore, and represents over 10% of the international study.

## 2 Methods

Full details of the ULIS-II (NCT01020500) methodology have been previously described (10). Briefly, ULIS-II was an observational, prospective, longitudinal cohort study following 468 adult patients (≥18 years old) with post-stroke upper limb spasticity over one BoNT-A injection cycle. All patients had to be treated with BoNT-A, but the decision to treat was taken independently from the decision to offer enrolment to the patient for participation in the study, and patients could be treated with any locally approved BoNT-A product.

Recruitment took place between January 2010 and May 2011. Clinicians treated patients in their normal way, guided by their local Product Information Documents and local therapeutic guidelines at the time of the study. The study was conducted in compliance with Guidelines for Good Pharmacoepidemiology Practices (GPP). Ethical approval and written informed consent to the recording of anonymous data was obtained at each participating site.

The primary objective was to assess the responder rate (as defined by the achievement of the primary goal from GAS) following one BoNT-A injection cycle. Achievement of primary and secondary GAS goals were rated at the end of the treatment cycle (timing per investigator judgment) on a six-point verbal rating scale and transcribed within the computer software to the five-point numerical scale (range − 2 to +2), and the GAS T score (10). Other measures of effectiveness included reduction in Modified Ashworth Scale (MAS) scores and Clinical Global Impression of Change (CGI-C). Secondary objectives were to describe patient characteristics (including demographics, duration, and pattern of spasticity) as well as concomitant therapies/medication and injection practices (e.g., muscle identification, dosage, dilution).

### 2.1 Statistical analysis

*Post-hoc* data are presented for all Asian patients (treated at 11 neurology and physiatry centers in Hong Kong, Philippines, Thailand, Taiwan, Malaysia, and Singapore) who received ≥1 BoNT-A injection and who underwent a post injection visit including an assessment of GAS. The primary outcome in the overall study and in this subgroup analysis was the responder rate (proportion of patients with primary goal GAS score 0, 1, or 2) following one BoNT-A injection cycle.

The vast majority (94.1%) of patients in the Asian subgroup were treated with abobotulinumtoxinA and, because units of BoNT-A dosing are non-interchangeable, injection data are presented for abobotulinumtoxinA-treated patients only. To provide context, descriptive regional data are presented alongside previously published results from the overall study (10); no formal statistical comparisons were made.

## 3 Results

### 3.1 Patient characteristics

Overall, 51 patients from Asia were enrolled and underwent ≥1 BoNT-A injection cycle, all of whom completed the study and were included in the analysis of effectiveness. Baseline characteristics for the Asian population alongside the international cohort are provided in Table 1. Of note, the mean ± SD time since the onset of stroke at the time of inclusion was 43.9 ± 66.1 months in the Asian subgroup, while in the global population it was 61.4 ± 69.1 months (10). There were fewer infarcts (56.9% vs. 70.2%) and more hemorrhagic strokes (43.1% vs. 30.5%) in the Asian subpopulation than in the global population. Asian patients presenting for routine treatment with BoNT-A were less likely than the international cohort to have impaired communication (15.7% vs. 34.9%), emotional regulation (15.7% vs. 32.7%), or impaired cortical function (5.9% vs. 13.2%). Rates of pain (3.9% vs. 17.1%) were also lower in the Asian subgroup, and while no Asian patients reported fatigue, it was reported in 10.5% of the international cohort (10).

**Table 1:** Patient characteristics at baseline.

	Asian subgroup (n = 51)	International cohort (*n* = 456) (10)
Age (years), mean (range)	57.1 (47–68)	56.7 (18–88)
Sex (male %), (M:F)	34 (66.7%), (2:1)	266 (58.3%), (3:2)
Time since onset of stroke (months), mean ± SD	43.9 ± 66.1	61.4 ± 69.1
Time since onset of spasticity (months), mean ± SD	27.9 (25.0)	48.2 (49.9)
First administration of BoNT-A in upper limb	28 (54.9%)	149 (32.7%)
Etiology of cerebrovascular accident (CVA)		
Infarct	29 (56.9%)	320 (70.2%)
Hemorrhage	22 (43.1%)	139 (30.5%)
Infarct and Hemorrhage	0	3 (0.7%)
Presence of mixed contractures	13 (25.5%)	116 (25.4%)
Presence of severe weakness	17 (33.3%)	264 (57.9%)
Impaired sensation, n (%)	22 (43.2%)	228 (50.0%)
Impaired communication, n (%)	8 (15.7%)	159 (34.9%)
Impaired cortical function, n (%)	3 (5.9%)	60 (13.2%)
Emotional/ behavioral dysfunction, n (%)	8 (15.7%)	149 (32.7%)
Presence of pain	2 (3.9%)	78 (17.1%)
Presence of fatigue	0	48 (10.5%)

In terms of motor presentation, patients presenting for routine BoNT-A treatment in Asia tended to have more severe distal limb spasticity (more distal joints with MAS score of ≥2), were less likely to have soft tissue shortening in the shoulder, and were less likely to show total loss of proximal function than those presenting for routine BoNT-A treatment internationally (3.9% vs. 20.8%) (10) (Supplementary Table S1).

### 3.2 Treatment for upper limb spasticity

Most (94.1%) patients in the Asian subgroup were treated with abobotulinumtoxinA (vs. 70.4% in the international cohort (10)); the three additional patients (5.9%) were treated with onabotulinumtoxinA. While the mean number of injected muscles was similar (5.5 vs. 5.0), total abobotulinumtoxinA dosing was lower in the Asian subgroup (mean 675.2 units; median 500 units) vs. the international cohort (mean 748.3 units; median 700 units); no patient in the Asian subgroup exceeded the maximum recommended dose of 1,000 units (Table 2). The patterns of muscles injected in the Asian subgroup generally reflected the different patterns of spasticity recorded in this subgroup (i.e., greater distal severity). In the Asian abobotulinumtoxinA subgroup, the most frequently injected muscles were the biceps brachii (83.3%), flexor carpi radialis (72.9%), and flexor digitorum profundus (66.7%). Use of injection guidance was lower in the Asian abobotulinumtoxinA subgroup (electromyography: 18.8%, electrical stimulation 12.5%) than in the international cohort (EMG: 28.3%, electrical stimulation 45.2%) (10). Six patients (11.8%) received concomitant BoNT-A therapy for another indication (e.g., lower limb spasticity).

**Table 2:** Botulinum toxin injection parameters (abobotulinumtoxinA treated patients).

	Asian subgroup (*n* = 48)	International cohort (*n* = 321)
Number of injected muscles; mean (SD)	5.5 (2.4)	5.0 (2.1)
Dose (units); mean (SD)	675.2 (289.2)	748.3 (333.2)
n/ n miss	48/0	321/0
Min, Max	80, 1,000	40, 1900
Median (IQR)	500.0 (500.0)	700.0 (500.0)
EMG used for at least one muscle	9 (18.8%)	91 (28.3%)
Electrical stimulation used for at least one muscle	6 (12.5%)	145 (45.2%)
Most commonly injected muscles		
Forearm and hand		
Flexor digitorum profundus	32 (66.7%)	180 (56.1%)
Flexor digitorum superficialis	29 (60.4%)	223 (69.5%)
Flexor pollicis longus	21 (43.8%)	95 (29.6%)
Adductor pollicis	8 (16.7%)	29 (9.0%)
Wrist		
Flexor carpi radialis	35 (72.9%)	188 (58.6%)
Flexor carpi ulnaris	28 (58.3%)	160 (49.8%)
Elbow		
Biceps brachii	40 (83.3%)	187 (58.3%)
Brachioradialis	20 (41.7%)	122 (34.9%)
Pronator teres	13 (27.1%)	102 (31.8%)
Brachialis	5 (10.4%)	77 (24.1%)
Shoulder		
Pectoralis major	14 (29.2%)	63 (19.6%)

Rates of physiotherapy in association with the BoNT-A treatment were similar for the Asian subgroup vs. the international cohort (58.9% vs. 60.7%) (Supplementary Table S2). By contrast, in line with the Asian focus treating the hand and fingers, rates of occupational therapy were higher in the Asian subgroup than in the international cohort (60.8% vs. 38.6%). Use of anti-spastic medication (e.g., baclofen) was higher in the Asian subgroup compared to the international cohort (41.2% vs. 28.5%).

### 3.3 Assessment of effectiveness

**Figure 1:**
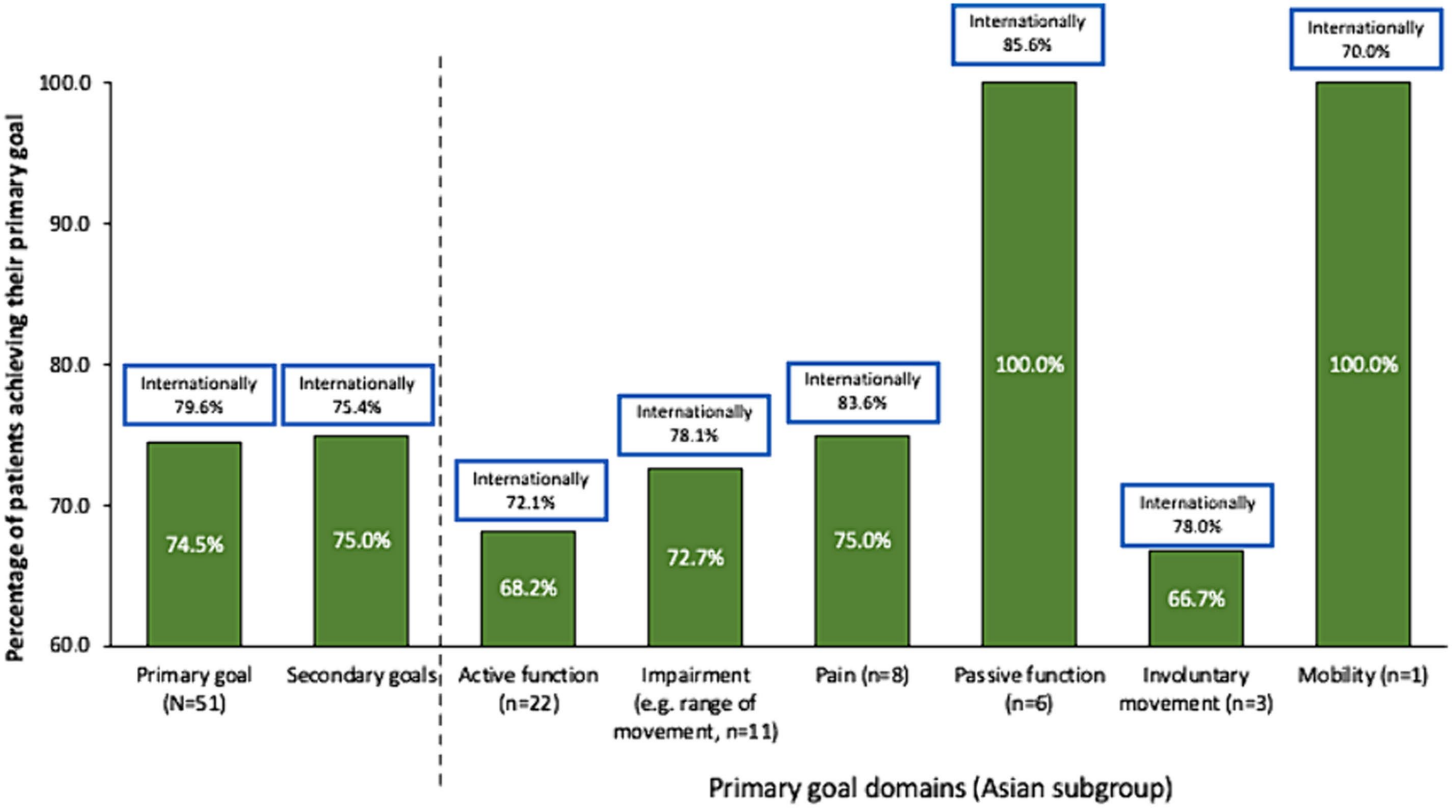
Goal achievement over 1 treatment cycle. Data in bars are for the Asian subgroup with international data in boxes for context. Goal achievement defined as a GAS score of 0, 1, or 2 at end of treatment cycle.

Whereas more patients in the international study set passive function goals than active function goals, Asian patients appeared to prioritize active function (Table 3). Rates of overall primary (74.5% vs. 79.6%, respectively) and secondary (75.0% vs. 75.4%) goal achievement were similar for the Asian group compared with the international cohort (10) (Figure 1). Overall goal attainment (as assessed by mean ± SD GAS T scores) was higher in the Asian subgroup compared to the international cohort (56.0 ± 13.0 vs. 52.0 ± 10.1), with Asian patients showing a change from baseline of 20.9 ± 14.3 (*p* < 0.001).

**Table 3:** Goals setting.

Goal areas	Asian subgroup (*n* = 51)	International cohort (*n* = 456)
Primary goals		
Pain	8 (15.7%)	61 (13.4%)
Passive function (ease of care)	6 (11.8%)	132 (29.0%)
Active function	22 (43.1%)	104 (22.8%)
Mobility (balance, gait)	1 (2.0%)	10 (2.2%)
Involuntary movement	3 (5.9%)	41 (9.0%)
Impairment	11 (21.6%)	105 (23.0%)
Other	0	3 (0.7%)
Secondary goals		
Pain	15 (40.5%)	83 (23.5%)
Passive function (ease of care)	8 (21.6%)	106 (30.0%)
Active function	8 (21.6%)	71 (20.1%)
Mobility (balance, gait)	2 (5.4%)	19 (5.4%)
Involuntary movement	2 (5.4%)	53 (15.0%)
Impairment	9 (24.3%)	102 (38.9%)
Other	0	5 (1.4%)

Reductions in muscle hypertonia (assessed by change from baseline in MAS scores) higher in the Asian subgroup vs. the international cohort (mean ± SD change of −4.3 ± 2.7 vs. −2.6 ± 2.6). According to the CGI-C, 96.1% of Asian patients were rated as having improved with BoNT-A treatment in the upper limb and 2 patients (4.2%) were rated as having no change (vs. 82.7% improved/ 8.3% no change / 1.0% worsened in the international cohort).

## 4 Discussion

Findings from this subgroup analysis confirm good response rates for patients living with upper limb spasticity treated with BoNT-A injection as delivered in Asian settings. While rates of goal attainment were comparable for patients presenting for routine BoNT-A treatment in Asian countries and the rest of the world (10), several findings from this *post-hoc* analysis highlight important differences in the patient population treated and in treatment parameters such as lower dosing and lower use of injection guidance. A lay summary of this subgroup analysis is provided in Supplementary material 2.

The relatively higher proportion of hemorrhagic stroke in the Asian subgroup is in line with the accumulating evidence for a higher proportion of intracerebral hemorrhage in patients with Chinese heritage versus white populations (11). However, it must be noted that there is also significant variation across Asia with studies in both rural (low-income) China (12) and Hong Kong (13) showing increases in rates of age-adjusted hemorrhagic stroke, while other studies report a marked reduction in hemorrhagic stroke in urban (higher income) China (14) and Taiwan (15). Whether or not the differences in Asian vs. international etiologies account for the differences in upper limb spasticity presentation between the two populations (e.g., greater distal severity in the Asian subgroup) is unclear. Lesion size in patients with hemorrhagic stroke is generally larger than in patients with ischemic stroke (16) and lesion volume is positively correlated with the severity of spasticity (17). However, it is also possible that the data reflect a referral bias of patients with distal (especially hand and fingers) spasticity to neurorehabilitation. Indeed, the relatively low reporting of comorbid communication and emotional problems in the Asian subgroup [e.g., versus the international cohort (10)] may indicate that patients with a less severe comorbidity profile are preferentially referred for upper limb rehabilitation.

BoNT-A injection parameters were in line with the patterns of spasticity reported, with the most commonly injected muscles being in the hand and finger flexors. The most widely used BoNT-A was abobotulinumtoxinA, which may partially reflect the injectors’ previous familiarity with the drug in previous Asian studies of minimal effective dosing (18) and impact of early treatment (within 12-weeks of the stroke event) (19). Other factors such as pricing per vial, potential for wastage with other products, and educational support may have also played a role in this choice. While the smaller muscle volumes (e.g., of the distal muscles) and the generally less severe presentation might contribute to the total lower doses used compared with the international study (10), the current data do suggest a general tendency for lower dosing in Asian vs. Western populations that goes beyond that expected due to the typically smaller stature and size of Asian vs. Western populations. Indeed, Asian studies have shown that patients who are treated earlier often require lower dosing (with less frequency) (19) and thus the earlier treatment seen in this subgroup may have also contributed to the lower dosing. Likewise, the emphasis on achieving active hand function goals might have led to lower dosing if injectors were concerned that higher doses might lead to weakness. Countries with hot climates may consider lower dosing strategies as a result of high temperature effects on muscle relaxation (18, 20). The maximum recommended dose of abobotulinumtoxinA in the upper limb is 1,000 units in any given treatment session (21) and while injectors in the international study appeared comfortable in using higher doses (10), the present data indicated that Asian clinicians are more likely to adhere to such guidelines. This may also reflect the reimbursement policies in each country, however there is significant variability in private versus national provision in the six countries included in this subgroup analysis (e.g., there is national reimbursement in Hong Kong, Malaysia, and Singapore but not in Thailand or the Philippines, and reimbursement strategies widely differ). Another significant difference in injection technique was the relatively low use of guidance techniques in Asian countries versus the international study. While it is generally well accepted that the effectiveness of the BoNT-A intervention is improved when using a guidance technique (22, 23), the lower use in Asia may reflect disparities in training, equipment availability and financial implications.

It is increasingly accepted that spasticity care should be goal oriented (24, 25), and the results of this study support the utility of goal attainment scaling as a practical way to assess the effectiveness of BoNT-A injections (26). Our findings indicate good rates of overall goal achievement in the Asian subgroup with clinically relevant (27) improvements in GAS T scores over one treatment cycle. In contrast to the international cohort, patients in the Asian subgroup tended to prioritize active over passive function, which may reflect the differences in patient presentation (e.g., distal vs. proximal spasticity), other factors (e.g., cultural), or a bias to refer patients who might be capable of achieving active function improvement to rehabilitation services. The most common goal statements for active function were related to being able to use the hand better in daily activities (e.g., better grip of the steering wheel during driving, the independent use of a spoon, and the ability to grasp and hold a glass). Of interest, while the shoulder is vital for many activities of living, including those requiring reach, the pectoralis major was only injected in <30% of patients and no patient received an injection into the subscapularis. A similar reluctance to inject the shoulder was seen at the international level (10), however more recent results from the ULIS-III study have since shown an increase in shoulder injections (28)—perhaps reflecting the recent increased education on the relevance of injecting these muscles (for goals beyond shoulder pain) (29).

As in the overall study, rates of active function achievement were lower than for other goal domains but were achievable for a significant proportion (68.2%) of patients. Such findings are important to highlight the role of BoNT-A injections in improving active functions in patients with the capacity for active function improvement. Recently, results from the ULIS-III study (which followed patients over several cycles) have indicated that patients with active function tended to require more frequent injections (up to 7 cycles in 2 years) (28). In this study, the good rates of active goal achievement following just a single injection might reflect several factors already discussed, such as the generally mild patient presentation, injector preference for early intervention as well as the high rates of concomitant occupational therapy. While the Asian sample size in this study is too small to reliably compare outcomes between subgroups, the overall international study showed that earlier treatment was associated with higher rates of active goal achievement, and that patients who received higher intensity therapy more likely to achieve their goal than those who received lower intensity therapy (30).

Strengths of this study include its multicenter, “real-world” nature which improves generalizability to clinical practice. However, it is important to acknowledge the limitations of conducting a *post-hoc* subgroup analysis of an open-label study. Most sites used abobotulinumtoxinA and thus no comparisons between brands can be made. In addition, while the Asian countries included in this subgroup share many common characteristics, we emphasize the diversity of several important factors such as national rates of hemorrhagic stroke as well key differences in health systems and access to reimbursement. The ULIS study program has continued since this study was conducted. Unfortunately, Asian representation in the ULIS III study was very small, and to our knowledge, these data from ULIS II remain the most robust Asian data on BoNT-A use available. More work to collect up to date local data is urgently needed.

Despite these limitations, the present data provide some of the first information about how BoNT-A is used to treat post-stroke upper-limb spasticity in Asian clinical practice. Our positive findings support the adoption goal-oriented spasticity care as the established protocol, emphasizing the benefits of early intervention. Moreover, they confirm that BoNT-A used within an integrated approach to spasticity management is effective in helping patients meet their functional treatment goals, thereby improving the daily life of patients living with post-stroke spasticity.

## Data availability statement

The original contributions presented in the study are included in the article/Supplementary material, further inquiries can be directed to the corresponding authors.

## Ethics statement

The study was conducted in compliance with Guidelines for Good Pharmacoepidemiology Practices. Marketing authorization for the use of BoNT-A in this context was ensured for each participating country prior to the start of the study. Ethical approval and written informed consent to the recording of anonymous data were obtained in countries where this was required. The studies were conducted in accordance with the local legislation and institutional requirements. The participants provided their written informed consent to participate in this study.

## Author contributions

RR: Conceptualization, Investigation, Project administration, Supervision, Visualization, Writing – original draft. NC: Conceptualization, Data curation, Funding acquisition, Project administration, Visualization, Writing – original draft. WK: Investigation, Writing – review & editing. KG: Investigation, Writing – review & editing. CM: Investigation, Writing – review & editing. KK: Investigation, Writing – review & editing. YN: Investigation, Writing – review & editing. LC: Investigation, Writing – review & editing. MF: Investigation, Writing – review & editing. TD: Project administration, Resources, Writing – review & editing. PM: Data curation, Formal analysis, Methodology, Resources, Validation, Visualization, Writing – review & editing. LL: Investigation, Writing – review & editing. AS: Conceptualization, Investigation, Supervision, Visualization, Writing – original draft, Writing – review & editing.
