# Supplementary material for: Botulinum toxin A injection for post-stroke upper limb spasticity and rehabilitation practices from centers across Asian countries
Source: Front Neurol. 2024 Apr 8;15:1335365. doi: 10.3389/fneur.2024.1335365 (PMC11034516; doi:10.3389/fneur.2024.1335365)
Supplement: Supplementary file 2 [file Data_Sheet_2.pdf]

Supplementary Material

1 Supplementary Data

Table e1. Motor presentation at baseline

	**Asian subgroup (n=51)**	**International cohort (n=456)**
Distribution of spasticity (MAS ≥ 2), n (%)		
Shoulder	26 (51.0%)	235 (51.5%)
Elbow	41 (80.4%)	335 (73.5%)
Wrist	44 (86.3%)	344 (75.4%)
Finger	44 (86.3%)	368 (80.7%)
Thumb	35 (68.6%)	292 (64.0%)
Soft tissue shortening (limiting ≥ ½ ranges), n (%)		
Shoulder	11 (21.6%)	156 (34.2%)
Elbow	17 (33.3%)	150 (32.9%)
Wrist	18 (35.3%)	179 (39.3%)
Hand	21 (41.2%)	206 (45.2%)
Motor function of the proximal upper limb (arm raising/ reaching)		
Normal: the subject is able to raise his/her arm above horizontal, normal power	4 (7.8%)	17 (3.7%)
Mild loss of motor function	15 (29.4%)	111 (24.3%)
Significant loss of motor function with some useful movement	30 (58.8%)	233 (51.1%)
No useful function	2 (3.9%)	95 (20.8%)
Motor function of the distal upper limb (hand function)		
Normal hand function	0	1 (0.2%)
Mild impairment of hand function, difficulty with fine motor control for manipulating small objects	5 (9.8%)	40 (8.8%)
Significant loss of motor function with some useful function (grasping/ stabilizing)	29 (56.9%)	155 (34.0%)
No useful function	17 (33.3%)	260 (57.0%)

Table e2. Concomitant treatments for South Asia and Global populations over one botulinum toxin cycle

	**Asia (n=51)**	**Global (n=456)**
Physiotherapy		
1-4 sessions	6 (20.0%)	33 (11.7%)
5-10 sessions	3 (10.0%)	50 (17.8%)
11-20 sessions	11 (36.7%)	74 (26.3%)
> 20 sessions	10 (33.3%)	120 (42.7%)
Unknown	0	4 (1.4%)
Occupational therapy		
1-4 sessions	7 (22.6%)	34 (18.9%)
5-10 sessions	4 (12.9%)	33 (18.3%)
11-20 sessions	13 (41.9%)	46 (25.6%)
>20 sessions	7 (22.6%)	63 (35.0%)
Unknown	0	4 (2.2%)
Splinting	17 (33.3%)	148 (32.5%)
Orthotics	10 (19.6%)	92 (20.2%)
Exercise	44 (86.3%)	360 (78.9%)
Passive stretching	42 (82.4%)	410 (89.9%)
Electrical stimulation	6 (11.8%)	58 (12.7%)
Positioning	24 (47.1%)	237 (52.0%)
Anti-spastic medication	21 (41.2%)	130 (28.5%)
